# Integrating Vertical Distribution, Quantitative Source Apportionment, and Source-Oriented Risk Assessment of Heavy Metals in Coastal Wetland Sediments: A Case Study from the Western Bohai Bay Watershed

**DOI:** 10.3390/toxics14080654

**Published:** 2026-07-25

**Authors:** Xinyi Lu, Gaohui Liu, Lixiao Wu, Runzhi Cui, Bao Xiang, Hongliang Wang, Honghai Xue

**Affiliations:** 1 State Key Laboratory of Environmental Criteria and Risk Assessment, Chinese Research Academy of Environmental Sciences, Beijing 100012, China; 2 Research Institute for Environmental Innovation (Binhai, Tianjin), Tianjin 300450, China; 3 Guangdong-Hong Kong-Macao Center for Eco-Environmental Sciences, Guangzhou 510555, China; 4 Key Laboratory of Songliao Aquatic Environment, Ministry of Education, Jilin Jianzhu University, Changchun 130118, China

**Keywords:** coastal wetland, heavy metals, vertical distribution, pollution characteristics, source apportionment, ecological and human health risk, source-oriented risk assessment

## Abstract

Coastal wetlands are important sinks for heavy metals, yet the linkage between vertical redistribution, quantitative source contributions, and ecological–health risk drivers remain insufficiently understood. This study collected the stratified sediment samples from three depth intervals (0–20, 20–40, and 40–60 cm) from 28 representative sites in the coastal wetlands of western Bohai Bay, and evaluated the spatial distribution, vertical variation, pollution status, potential sources, and ecological–human health risks of eight heavy metals (Cr, Ni, Cu, Zn, As, Cd, Hg, and Pb). The mean concentrations were below the Class I limits of the Marine Sediment Quality Standard. Most metals were close to or slightly below regional background values, whereas Cu and As showed mild enrichment and Cr was higher than values reported for Bohai Bay and Hangzhou Bay. Vertical profiles were generally homogeneous, with weak enrichment of specific metals, probably due to sediment resuspension, tidal disturbance, and bioturbation. Pollution assessment based on the geoaccumulation index (Igeo), Nemerow pollution index (P_N_), and pollution load index (PLI) consistently indicated low contamination levels, with Hg and Pb as the main contributors to the regional pollution load. Source apportionment indicated that heavy metals were jointly influenced by lithogenic background (38.9%), atmospheric deposition (31.1%), and local anthropogenic activities (30.0%). Ecological risk assessment showed that the integrated risk index (RI) remained within the low-risk category, although Hg consistently fell within the moderate-risk range and Cd approached the moderate-risk threshold. Health risk assessment showed that both non-carcinogenic and carcinogenic risks were within acceptable limits for all receptor groups. Children had higher risks in core residential areas, whereas adults showed higher risks in non-core industrial–agricultural zones because of increased exposure frequency. The source–risk analysis indicates that non-carcinogenic risk is mainly associated with background-derived Cr, whereas carcinogenic risk is primarily linked to anthropogenic As inputs. These findings indicate that source contributions and risk contributions are not necessarily consistent, highlighting the need for source-oriented risk management in industrialized coastal wetlands.

## 1. Introduction

Coastal wetlands are ecologically sensitive transition zones between terrestrial and marine environments. They provide important ecosystem functions, including biodiversity conservation, carbon sequestration, and the retention of land-derived contaminants [1,2,3]. Owing to their strong adsorption capacity and long-term accumulation potential, wetland sediments serve as major sinks for heavy metals and effectively record both natural geochemical processes and anthropogenic disturbances [3,4,5]. With increasing industrialization, urbanization, and port development in coastal regions worldwide, heavy metal contamination in wetland sediments has become a growing environmental concern because of its persistence, bioaccumulation potential, and ecological toxicity [6,7,8,9]. Furthermore, sediments may act as secondary pollution sources when environmental conditions change, leading to the remobilization of contaminants and posing potential risks to aquatic ecosystems and human health through trophic transfer and long-term exposure. Recent studies have advanced our understanding of heavy metal contamination in coastal and estuarine sediments. Spatial enrichment patterns have been widely reported in the Yangtze River Delta, Pearl River Estuary, Yellow River Delta, and other coastal regions, and industrial emissions, shipping activities, atmospheric deposition, and agricultural runoff have been identified as important sources [10,11,12]. Similarly, studies in the Mediterranean Sea have demonstrated that heavy metal contamination in coastal sediments is closely linked to anthropogenic activities and insufficiently treated wastewater discharges, with concentrations decreasing from nearshore discharge areas toward the open sea [13]. Source apportionment has commonly been conducted using multivariate statistical methods, such as principal component analysis (PCA), hierarchical cluster analysis (HCA), and positive matrix factorization (PMF) [14,15,16]. Meanwhile, ecological risk indices and human health risk models have been increasingly applied to evaluate the potential impacts of sediment-associated heavy metals [17,18,19]. However, many studies still treat spatial distribution, source apportionment, and risk assessment as separate analytical steps, which limits a mechanistic understanding of how source contributions are transformed into ecological and health risks [20]. Despite these advances, three key scientific questions remain insufficiently resolved. First, vertical redistribution processes of heavy metals in tidally influenced coastal wetland sediments remain poorly characterized, making it difficult to distinguish surface enrichment, profile homogenization, and potential remobilization processes [20]. Second, quantitative source apportionment has rarely been explicitly linked to ecological and human health risk outcomes, limiting the identification of source-specific risk drivers [21,22]. Third, it remains unclear whether the dominant contributors to total metal loads are also the dominant contributors to ecological or health risks under complex multi-source conditions [23]. These gaps are particularly important for industrialized coastal wetlands, where tidal mixing, sediment resuspension, multiple anthropogenic inputs, and heterogeneous human exposure scenarios may jointly regulate metal behavior and risk expression [24]. Previous studies on heavy metals in Bohai Bay have primarily focused on surface sediment contamination in the central bay and adjacent estuaries. Gao and Chen [25] reported elevated Pb and Zn concentrations in surface sediments of coastal Bohai Bay, attributing them to riverine inputs and atmospheric deposition. Li et al. [26] assessed the ecological risks of heavy metals in Bohai Sea sediments and identified Cd and Hg as the primary risk drivers. More recently, regional comparisons have revealed that heavy metal concentrations in Bohai Bay sediments vary considerably across different coastal zones, reflecting the combined influence of natural sedimentary processes and localized anthropogenic activities [27,28,29]. Our previous work [30] examined the spatial distribution and contamination levels of heavy metals in surface sediments (0–20 cm) of the TBNA coastal wetlands, identifying Hg as the primary ecological risk contributor and revealing high spatial variability of Cd, Hg, and Pb associated with port-industrial activities. However, that study was limited to surface sediments and did not quantify source contributions or evaluate human health risks. Most existing studies have been limited to the surface layer (0–5 cm) and have rarely examined the vertical redistribution of heavy metals in tidally influenced coastal wetlands. Furthermore, the quantitative linkage between source contributions and ecological–health risks has not been systematically addressed in this region. These gaps highlight the need for an integrated assessment that combines vertical distribution analysis, source apportionment, and source-oriented risk evaluation in the coastal wetlands of western Bohai Bay.

To address these gaps, this study established an integrated framework linking vertical distribution characteristics, quantitative source apportionment, and ecological–health risks of heavy metals in coastal wetland sediments. Stratified sediment samples were collected from three depth intervals (0–20, 20–40, and 40–60 cm) at 28 representative sites along the western coast of Bohai Bay. The specific objectives were to: (i) characterize the spatial and vertical distribution patterns of eight heavy metals using statistical analysis and the vertical distribution index (VDI); (ii) evaluate contamination levels using multiple pollution assessment indices; (iii) quantify lithogenic, atmospheric, and anthropogenic source contributions using an integrated source-identification framework combining correlation analysis, HCA, PCA, and PMF; (iv) assess ecological risks using the Hakanson potential ecological risk index and identify key ecological risk contributors; (v) evaluate probabilistic human health risks under different receptor groups and exposure scenarios using Monte Carlo simulation; and (vi) integrate source apportionment with ecological and health risk results to identify source-related risk drivers. The results contribute to a better understanding of how source contributions are translated into ecological and human health risks and provide a transferable framework for source-oriented risk management in industrialized coastal wetland systems.

## 2. Materials and Methods

### 2.1. Study Area

The study area is located at Tianjin, along the western coast of Bohai Bay (38°40′–39°00′ N, 117°20′–118°00′ E). This region is one of the most intensively developed coastal zones in northern China and is influenced by multiple anthropogenic activities, including port logistics, petrochemical production, industrial manufacturing, urban expansion, and agricultural development. Coastal wetlands in this region receive land-derived inputs through river discharge, surface runoff, atmospheric deposition, and port-related activities. The coexistence of tidal disturbance, sediment resuspension, and multiple pollution sources makes this area a representative industrialized coastal wetland system for investigating the vertical distribution, source contributions, and ecological–health risks of sediment heavy metals. The study area is underlain by fine-grained sediments (silt and clay) derived from fluvial and marine interactions. Such fine-grained sediments are conducive to heavy metal adsorption due to their large specific surface area and high organic matter content.

### 2.2. Sample Collection and Laboratory Analysis

**Figure 1:**
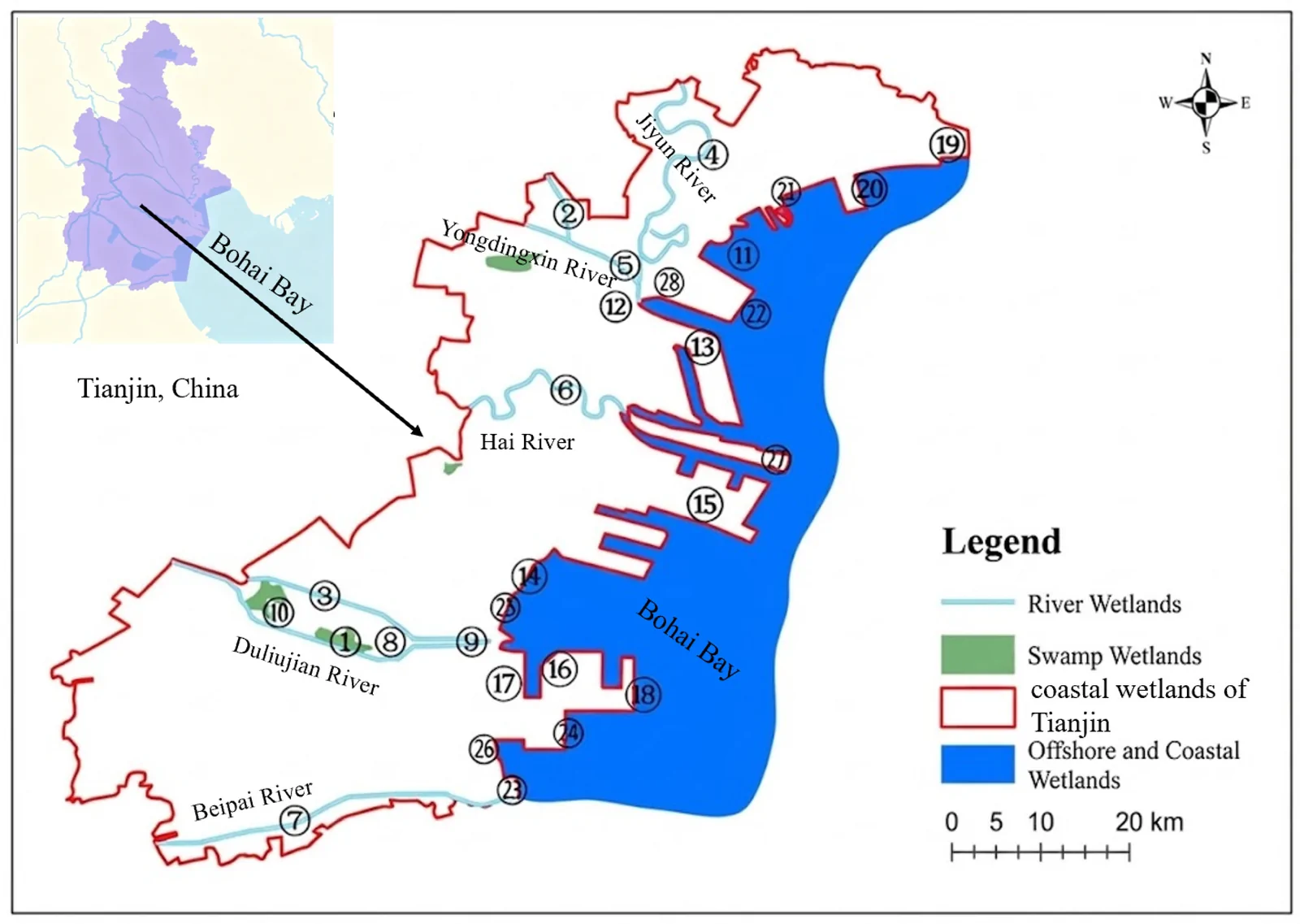
Location of the study area and distribution of sediment sampling sites (*n* = 28) in the coastal wetlands of Tianjin, China.

Sediment cores were collected at 28 representative sites in the study area in April 2025 (spring season), covering 14 sites in coastal wetlands/intertidal zones and 14 sites in riverine wetlands near major river estuaries, using a stainless-steel column sampler and sectioned into three depth intervals: 0–20, 20–40, and 40–60 cm. A core depth of 60 cm was selected to capture both recent and historical deposition signals, while the 20 cm interval was chosen to balance vertical resolution with analytical feasibility. The sampling sites covered industrial-dominated, port-logistics, and agricultural-influenced areas, as shown in Figure 1. Samples were stored at −20 °C before pretreatment, freeze-dried, ground, and passed through a 200-mesh sieve. All digestion and heavy metal measurement procedures were completed by a qualified commercial testing laboratory. For metal analysis, 0.25 g of dried sediment powder was digested with an HNO_3_–HF–HClO_4_ mixture (5:3:2, *v*/*v*/*v*) using a programmed microwave digestion procedure: 120 °C for 10 min, 160 °C for 20 min, and 180 °C for 30 min. Concentrations of eight heavy metals (Cr, Ni, Cu, Zn, As, Cd, Hg, and Pb) were determined using ICP-MS (Agilent 8900, Santa Clara, CA, USA) in helium collision mode. Quality control was performed using certified reference material GBW07314a. The recoveries ranged from 94.2% to 107.8% (*n* = 5), and the relative standard deviations were below 5%. Method detection limits were determined according to HJ 803–2016 [31].

### 2.3. Analysis of Sediment Physicochemical Properties

Sediment pH was measured potentiometrically at a solid-to-liquid ratio of 1:2.5. Moisture content (SMC) was determined by the gravimetric method, bulk density (SBD) by the cutting-ring method, and organic carbon (OC) and total nitrogen (TN) by an elemental analyzer. Total phosphorus (TP) was determined by the acid digestion–molybdenum antimony anti-spectrophotometric method. Quality assurance included method blanks, duplicate samples, and standard reference materials, with all deviations maintained within acceptable ranges.

### 2.4. Statistical Analysis

Descriptive statistics, including mean, median, standard deviation (SD), and coefficient of variation (CV), were calculated to characterize metal distributions. Prior to statistical analysis, data normality was assessed using the Shapiro–Wilk test. Inter-layer differences were assessed using one-way ANOVA followed by Tukey’s HSD test (*p* < 0.05). Since most heavy metal concentrations did not follow a normal distribution, non-parametric Spearman correlation analysis was applied. Source identification was further supported by Mantel tests, HCA, and PCA [32]. Statistical analyses and visualizations were conducted using Python 3.12, while PMF source apportionment was performed using EPA PMF 5.0.

### 2.5. Assessment Methods

#### 2.5.1. Vertical Distribution Analysis

Traditional vertical assessment methods often rely on simple concentration ratios or enrichment factors, which frequently fail to capture the complex relationships among multiple sediment layers [33]. To address this limitation, the vertical distribution index (VDI_i_) was employed to quantitatively characterize the surface enrichment and overall vertical distribution patterns of heavy metals within sediment profiles. The VDI_i_ is calculated as follows:

(1) $$\mathrm{VD}I_{i}=\frac{C_{i,s}+C_{i,m}}{C_{i,b}}$$

where VDI_i_ represents the vertical distribution index of metal i; and C_i,s_, C_i,m_, and C_i,b_ represent the concentrations of metal i in the surface (0–20 cm), middle (20–40 cm), and bottom (40–60 cm) layers, respectively. Generally, a VDI_i_ value below 3 indicates no pronounced enrichment in the upper sediment layers relative to the bottom layer. It should be noted that the VDI_i_ serves as a relative indicator of vertical distribution patterns rather than a quantitative measure of metal migration fluxes or source contributions.

#### 2.5.2. Pollution Indices Assessment

The geoaccumulation index (I_geo_), Nemerow pollution index (P_N_), and pollution load index (PLI) were used to evaluate sediment contamination [30].

The I_geo_ was calculated as follows [34,35]:

(2) $$I_{\mathrm{geo},i}=\log_{2}\left(\frac{C_{i}}{1.5\times B_{i}}\right)$$

where $C_{i}$ is the measured concentration of metal i, $B_{i}$ is the corresponding background value, and 1.5 is a correction factor accounting for natural lithogenic variability. The selection of appropriate background values is essential for accurate contamination assessment, as these values can vary considerably across different regions due to differences in natural geological conditions [36].

The Nemerow pollution index (P_N_) was calculated as [37,38]:

(3) $$P_{N}=\sqrt{\frac{P_{i,max}^{2}+P_{i,\mathrm{mean}}^{2}}{2}},P_{i}=\frac{C_{i}}{B_{i}}$$

where $P_{i}$ is the single-factor pollution index and $P_{i,max}$ and $P_{i,\mathrm{mean}}$ are the maximum and mean values of $P_{i}$ among the analyzed metals, respectively.

The pollution load index (PLI) was calculated as [39,40]:

(4) $$\mathrm{PLI}={\left(CF_{1}\times CF_{2}\times \cdots \times CF_{n}\right)}^{1/n},\;CF_{i}=\frac{C_{i}}{B_{i}}$$

where $CF_{i}$ is the contamination factor of metal i, and n is the number of metals. The classification criteria for I_geo_, P_N_, and PLI are provided in Table S1. To compare the relative contribution of individual metals to the regional pollution load, the CF of each metal was averaged across all sampling sites and sediment layers and visualized using a petal diagram.

#### 2.5.3. Source Apportionment

Heavy metal sources were identified using a sequential approach that integrated Spearman correlation analysis, hierarchical cluster analysis (HCA) [41], principal component analysis (PCA) [42], and positive matrix factorization (PMF) [43]. Spearman correlation analysis, HCA, and PCA were used to explore inter-element relationships and qualitatively infer potential source categories, including lithogenic background, atmospheric deposition, industrial emissions, and agricultural inputs. HCA was performed using Ward’s linkage method with Euclidean distance as the similarity measure. PCA was conducted with varimax rotation, and components with eigenvalues >1 were retained.

For quantitative source apportionment, the EPA PMF 5.0 model was applied [44]. The concentration matrix was decomposed according to the following bilinear model:

(5) $$x_{ij}=\sum_{k=1}^{p}g_{ik}f_{kj}+e_{ij}$$

where $x_{ij}$ is the concentration of species j in sample i, $g_{ik}$ is the contribution of factor k to sample i, $f_{kj}$ is the profile of species j in factor k, $e_{ij}$ is the residual, and p is the number of factors. The objective function was defined as:

(6) $$Q=\sum_{i=1}^{n}\sum_{j=1}^{m}{\left(\frac{e_{ij}}{u_{ij}}\right)}^{2}$$

where $u_{ij}$ is the uncertainty of species j in sample i. Uncertainties were calculated according to EPA PMF guidelines based on method detection limits and species-specific error fractions. Different factor solutions were tested, and the optimal solution was selected based on Q/Qexp, residual distributions, factor interpretability, and run stability.

#### 2.5.4. Ecological Risk Assessmentmethod

The potential ecological risk factor ($E_{r}^{i}$) and integrated ecological risk index (RI) were used to evaluate the ecological risks of heavy metals by combining contamination levels with metal-specific toxicity coefficients [45]. They were calculated as follows:

(7) $$RI=\sum_{i=1}^{n}E_{r}^{i}=\sum_{i=1}^{n}\left(T_{r}^{i}\times C_{f}^{i}\right)=\sum_{i=1}^{n}\left(T_{r}^{i}\times \frac{C_{i}}{C_{n}^{i}}\right)\;$$

where $C_{f}^{i}$ is the contamination factor of metal i, $C_{i}$ is the measured concentration, $C_{n}^{i}$ is the corresponding background value, $T_{r}^{i}$ is the toxic-response factor, $E_{r}^{i}$ is the potential ecological risk factor of metal i, and RI is the integrated ecological risk index for all analyzed metals. In this study, Chinese offshore sediment background values were used as reference values. The toxic-response factors were set as Hg = 40, Cd = 30, As = 10, Pb = Cu = Ni = 5, Cr = 2, and Zn = 1. The classification criteria for $E_{r}^{i}$ and RI are provided in Table S2. Chinese offshore sediment background values were used as reference values [46].

#### 2.5.5. Human Health Risk Assessment

Human health risks from sediment heavy metals were assessed for adults and children following the USEPA exposure model. Three exposure pathways were considered: ingestion, dermal contact, and inhalation [47,48,49]. The average daily doses (ADD, mg·kg^−1^·day^−1^) via ingestion, dermal contact, and inhalation was calculated as follows:

(8) $${\mathrm{ADD}}_{\mathrm{ing}}=\frac{C\times \mathrm{IR}\times \mathrm{EF}\times \mathrm{ED}}{\mathrm{BW}\times \mathrm{AT}}$$

(9) $${\mathrm{ADD}}_{\mathrm{dermal}}=\frac{C\times \mathrm{SA}\times \mathrm{AF}\times \mathrm{ABS}\times \mathrm{EF}\times \mathrm{ED}}{\mathrm{BW}\times \mathrm{AT}}$$

(10) $${\mathrm{ADD}}_{\mathrm{inh}}=\frac{C\times \mathrm{InhR}\times \mathrm{EF}\times \mathrm{ED}}{\mathrm{PEF}\times \mathrm{BW}\times \mathrm{AT}}$$

where $C_{i}$ is the concentration of metal i, IR is the ingestion rate, EF is the exposure frequency, ED is the exposure duration, BW is body weight, AT is the averaging time, SA is the exposed skin area, AF is the skin adherence factor, ABS is the dermal absorption factor, InhR is the inhalation rate, and PEF is the particle emission factor.

To improve the regional applicability of the risk assessment, several localized correction factors were incorporated. Exposure frequency was differentiated by land-use scenario and receptor group, with 90 d/y assigned to core residential areas, 250 d/y to adults in non-core industrial–agricultural areas, and 50 d/y to children in non-core areas, following a scenario-based exposure assessment framework [47]. For carcinogenic risk estimation, 1% of total Cr was assumed to occur as Cr(VI), consistent with previous exposure assessments using a 1% Cr(VI)-to-total-Cr assumption for soil or dust [50]. An oral bioavailability factor of 0.15 was applied to As, and the inhalation carcinogenic risk contribution was weighted by a local correction factor of 0.005.

Non-carcinogenic risk was evaluated using the hazard quotient (HQ) and hazard index (HI):

(11) $$HQ_{ij}=\frac{ADD_{ij}}{RfD_{ij}},\;HI=\sum_{i=1}^{n}\sum_{j=1}^{m}HQ_{ij}$$

where $RfD_{ij}$ is the reference dose of metal i through exposure pathway j. An HI value below 1 indicates no significant non-carcinogenic risk, whereas HI ≥ 1 indicates potential non-carcinogenic concern.

Carcinogenic risk was evaluated using the carcinogenic risk (CR) and total carcinogenic risk (TCR):

(12) $$CR_{ij}=ADD_{ij}\times SF_{ij},\;TCR=\sum_{i=1}^{n}\sum_{j=1}^{m}CR_{ij}$$

where $SF_{ij}$ is the carcinogenic slope factor of metal i through pathway j. A TCR value below $1\times 10^{-6}$ indicates negligible risk, $1\times 10^{-6}\leq TCR<1\times 10^{-4}$ indicates acceptable risk, and $TCR\geq 1\times 10^{-4}$ indicates potentially unacceptable carcinogenic risk.

To account for uncertainty and variability in exposure parameters, a Monte Carlo simulation with 100,000 iterations was performed [51,52]. Key parameters, including body weight, ingestion rate, and exposure frequency, were assigned probability distributions based on USEPA recommendations and local exposure scenarios. Detailed exposure parameters are provided in Table S3, and toxicity parameters and localized correction factors are provided in Table S4.

## 3. Results and Discussion

### 3.1. Sediment Physicochemical Properties

The physicochemical properties of the sediments are summarized in Table 1 The sediments were generally weakly alkaline, with a mean pH of 8.21 and minor spatial variation (CV < 0.03), indicating a stable alkaline environment favorable for heavy metal adsorption and precipitation. Mean SMC and SBD were 41.8% and 1.35 g·cm^−3^, respectively. OC averaged 7.06 g·kg^−1^, with slightly higher values in the upper and middle layers than in the bottom layer, suggesting surface enrichment associated with plant litter input. TN and TP were relatively low, with mean values of 0.062% and 0.062%, respectively. In terms of spatial variability, OC (CV: 0.515–0.597), SMC (CV: 0.452–0.465), and TN (CV: 0.438–0.551) exhibited strong variation, whereas pH and SBD showed weak-to-moderate variation. These results indicate that sediment physicochemical properties in the study area are markedly influenced by local micro-environments and external inputs, and collectively constitute the fundamental conditions governing heavy metal migration and accumulation in the sediments.

### 3.2. Spatial Distribution Characteristics of Heavy Metals

Heavy metal concentrations in the coastal wetland sediments were generally low and showed element-specific spatial variability. As shown in Table 2 the mean concentrations of Cr, Ni, Cu, Zn, As, Cd, Hg and Pb were 74.16, 26.94, 23.74, 71.05, 11.33, 0.14, 0.04, and 27.64 mg/kg, respectively. The coefficients of variation (CVs) indicated clear differences in spatial heterogeneity among metals. Cd (30.1%), Hg (31.5%), Pb (36.2%), and Cu (31.6%) showed relatively high variability, suggesting that their distributions were influenced by localized external inputs, such as port activities, industrial emissions, or atmospheric deposition. Contrastingly, Cr and Ni showed moderate variability, likely reflecting stronger lithogenic control, whereas the source characteristics of As and Zn required further multivariate verification. This pattern is consistent with coastal wetlands where natural sedimentary backgrounds are locally modified by human activities [53].

Comparison with regional background values provided further evidence for this pattern. Cr, Zn, and Cd were comparable to or slightly below background levels, whereas Pb was slightly higher than its background value [46]. Cu and As exceeded their respective background values, indicating mild enrichment that may be related to port–industrial activities, agricultural inputs, or sediment geochemical conditions in coastal transition zones. All mean metal concentrations were below the Class I limits of the Marine Sediment Quality Standard (GB 18668-2002) [54], indicating generally good sediment quality in the study area.

Cross-regional comparisons further contextualized the contamination status of the studied sediments. Cr concentrations in sediments were higher than those reported for Bohai Bay in 2015 and Hangzhou Bay in 2022 [28], but slightly lower than those reported for Haizhou Bay [29] (Table S5). This relatively high Cr level may be associated with metallurgical activities in the Lingang Industrial Zone and the retention of fine particles under local hydrodynamic conditions [25]. Cd levels were lower than historical values reported for Bohai Bay, suggesting weaker Cd enrichment in the present sediments. Pb levels were comparable to those in Haizhou Bay but showed high variability, suggesting localized enrichment potentially related to shipping activities and port operations [55]. Hg levels were comparable among the compared coastal systems, consistent with the influence of regional atmospheric deposition, particularly from coal combustion [56]. Cu, Zn, and As were generally lower than values reported for the other compared coastal regions. Overall, these comparisons indicate that the sediments are characterized by generally low contamination, with localized anthropogenic enrichment for specific metals.

### 3.3. Vertical and Inter-Layer Distribution Characteristics of Heavy Metals

**Figure 2:**
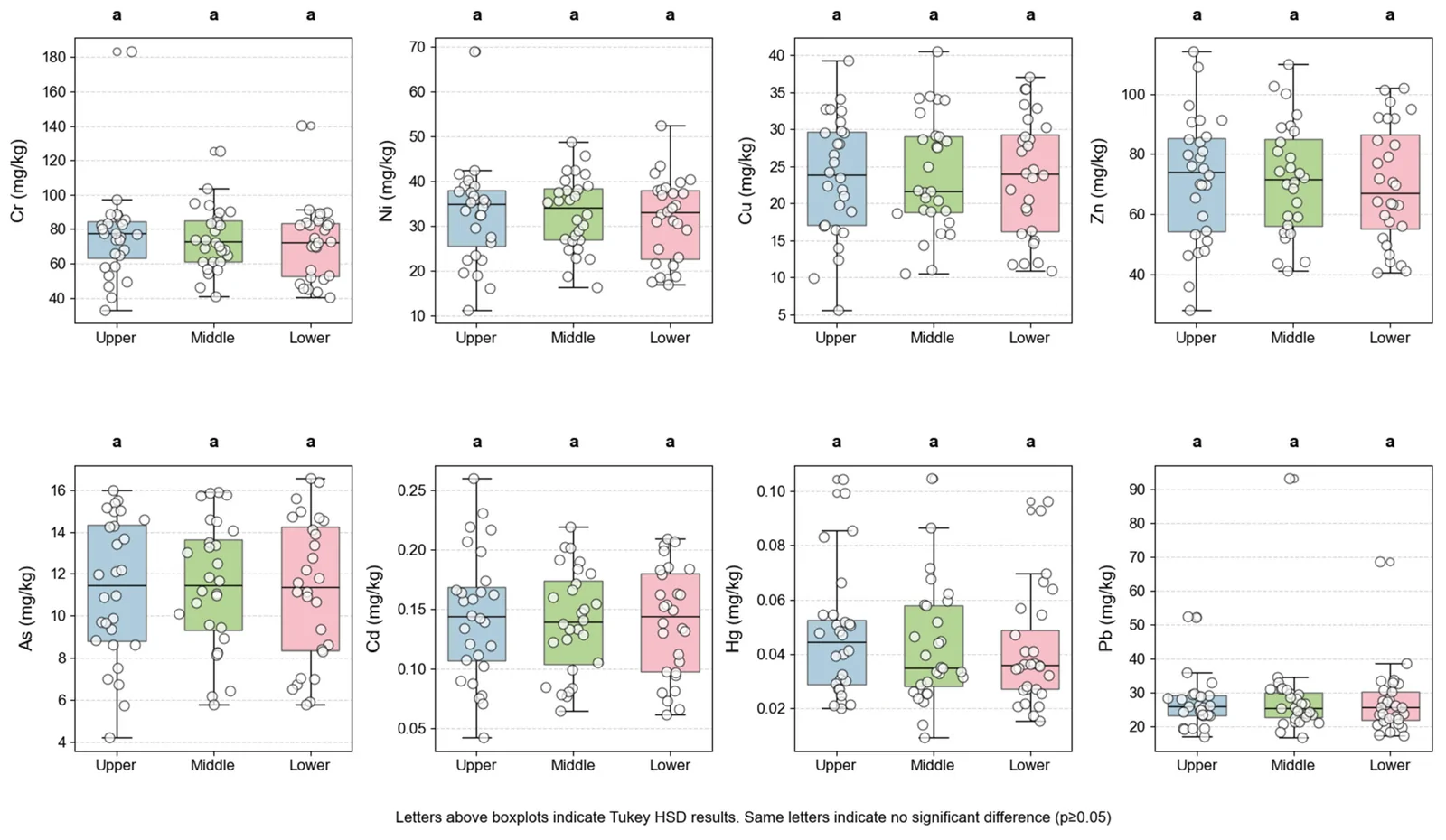
Average heavy metal concentrations across different layers. Note: The letters above the boxplots represent the results of the Tukey post-hoc test. Groups sharing the same letter are not significantly different (*p* > 0.05).

After characterizing the horizontal distribution, the vertical profiles were examined to evaluate whether heavy metals showed surface enrichment or depth-dependent accumulation. Heavy metal concentrations were compared among the surface (0–20 cm), middle (20–40 cm), and bottom (40–60 cm) layers. As shown in Figure 2, the concentrations of all metals were generally similar across the three sediment layers. Tukey’s HSD tests showed no significant inter-layer differences for any metal (*p* > 0.05), indicating no pronounced vertical stratification. This vertically uniform pattern differs from the surface enrichment reported in some estuarine and lacustrine sediments and may reflect intensive sediment reworking [57,58]. In the coastal wetlands, repeated resuspension–deposition cycles and tidal disturbance likely promote vertical mixing, while bioturbation may further contribute to the homogenization of metal concentrations within the sediment column [59].

**Figure 3:**
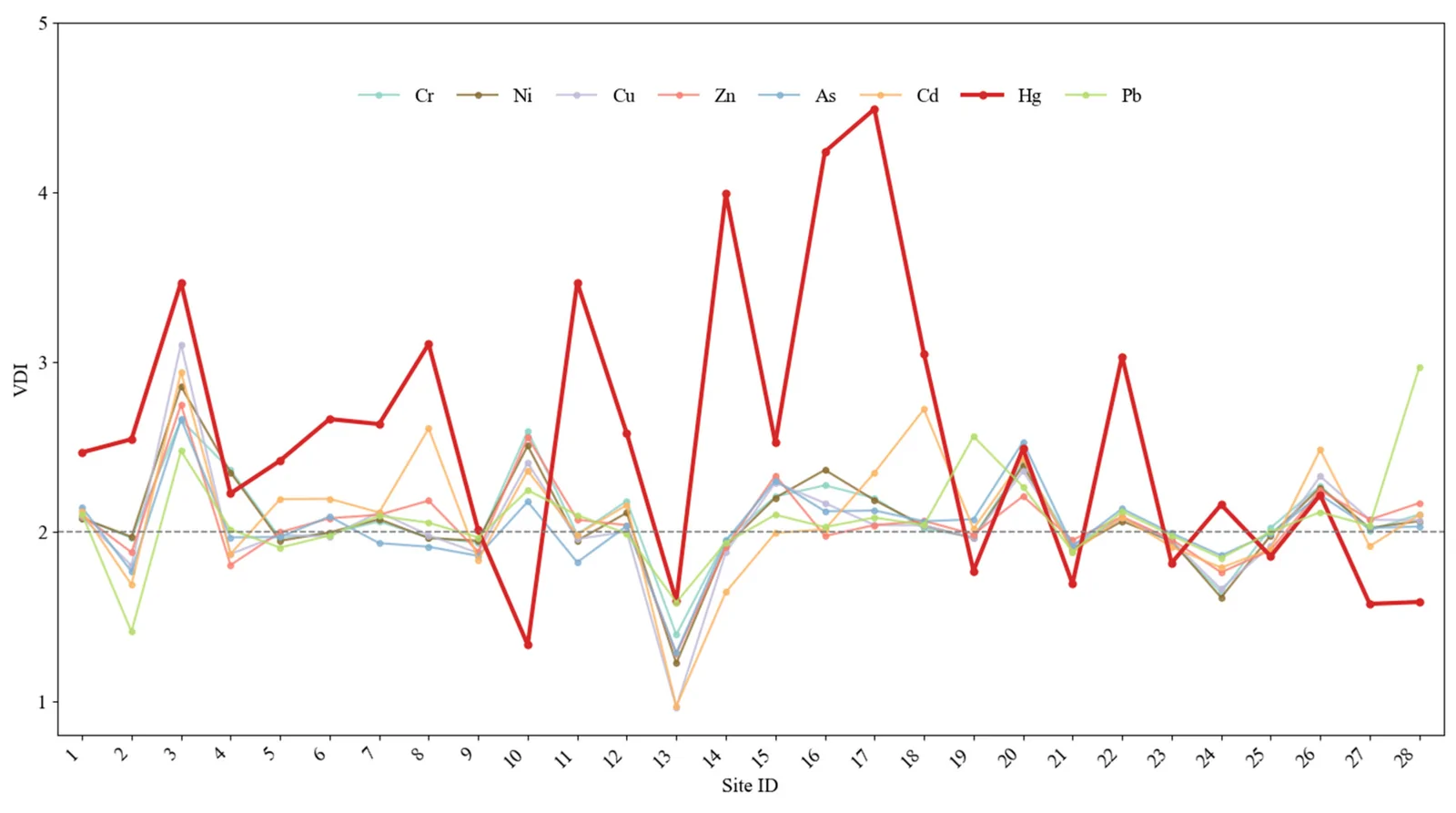
Results of interlayer distribution analysis.

To further characterize subtle vertical differences, the vertical distribution index (VDI) was applied. As shown in Figure 3, VDI values for most metals were mainly distributed around 2 across sampling sites, indicating generally homogeneous vertical distributions without consistent enrichment in the upper layers. This finding is consistent with the small inter-layer differences observed for most metals in similar coastal settings. Nevertheless, localized deviations were observed for certain metals. Hg exhibited elevated VDI values at several sites, exceeding 3 in some cases, suggesting relative enrichment in the upper sediment layers. Layer-averaged concentration differences further supported this interpretation. Pb showed the clearest mid-layer enrichment, as reflected by positive $\Delta C_{mid-surf}$ and $\Delta C_{mid-bottom}$ values (Table S6). Cu and As also showed weaker mid-layer accumulation, whereas Cr, Ni, and Zn exhibited relatively small inter-layer differences, indicating more stable vertical distributions.

Layer-specific coefficients of variation (CVs), calculated from layer-averaged concentrations (Table S7), further reflected differences in metal variability within the sediment column. Higher CVs for Hg, and to a lesser extent Cd, across the sediment layers suggest greater influence from localized external inputs and post-depositional redistribution. In contrast, the lower and more uniform CVs of Cr and Ni are consistent with stable geochemical backgrounds. Physicochemical conditions may partly explain these patterns. Sediment pH remained weakly alkaline with limited depth variation, which may constrain substantial metal mobilization. Sediment organic carbon (SOC) showed stronger vertical variability, with higher values generally occurring in the middle layer. This SOC pattern may enhance metal retention through adsorption and complexation, contributing to the observed mid-layer enrichment [60,61]. Overall, the heavy metals in sediments showed generally homogeneous vertical distributions, with weak enrichment of specific metals controlled by sediment mixing and depth-dependent physicochemical conditions.

### 3.4. Heavy Metal Pollution Assessment

**Figure 4:**
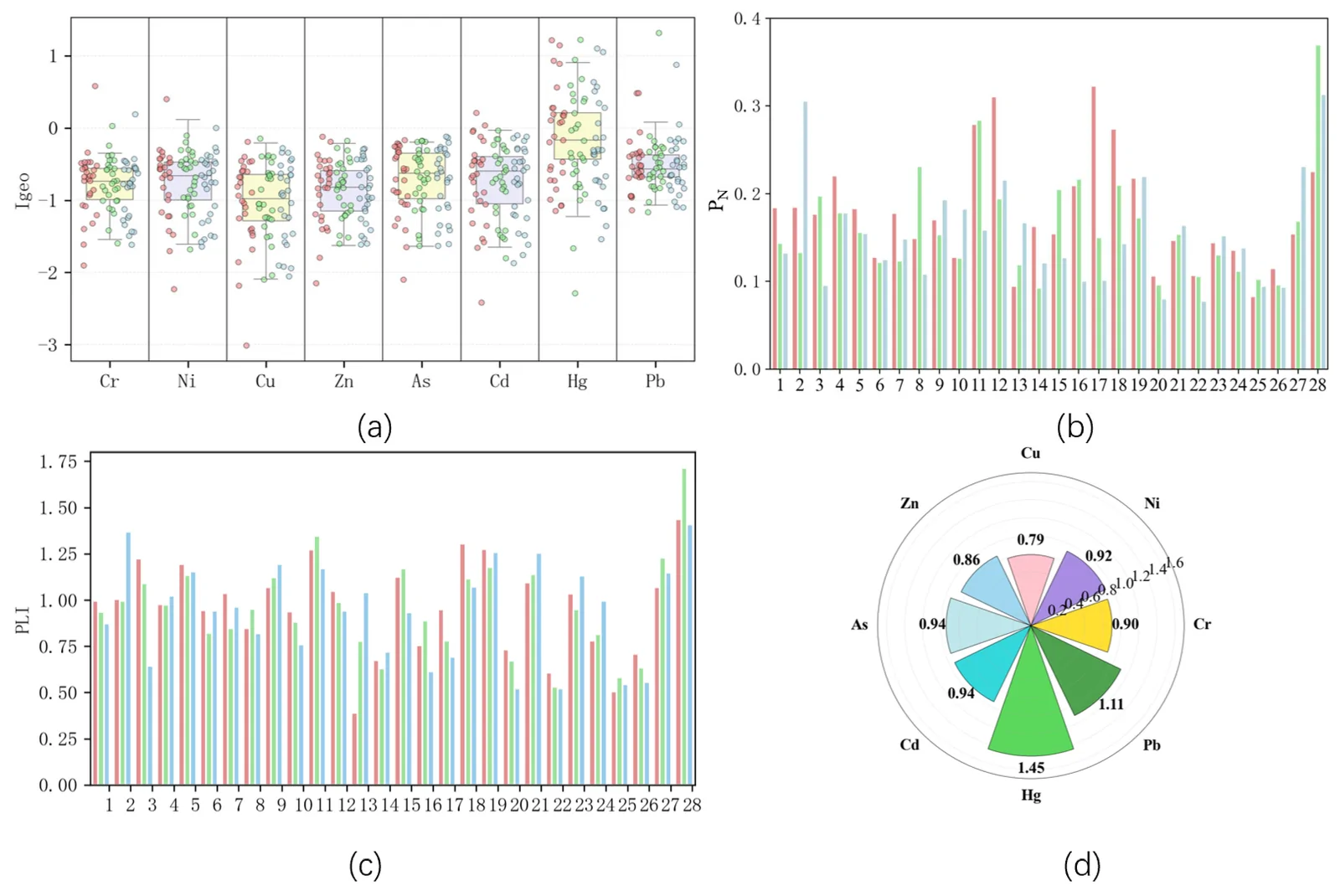
Spatial variation of heavy metal pollution indices in sediments of the study area. (**a**) Igeo box-scatter plots for heavy metals; (**b**) Spatial distribution of P_N_ (**c**) Spatial distribution of PLI; (**d**) Mean pollution contribution coefficients of heavy metals. Blue, red and green bars in (**a**–**c**) represent three sampling subgroups.

To represent the overall contamination status of each site, pollution indices were calculated using the mean concentrations across the three sediment layers. The results indicated that the Igeo values of most metals (Cr, Ni, Cu, Zn, As, Pb, and Cd) were mainly between −1 and 0, corresponding to an unpolluted status or values close to the unpolluted threshold (Figure 4a). By comparison, Hg showed a median Igeo value close to 0, and several sites exceeded Igeo = 0, indicating slight Hg enrichment and relatively stronger external influence. It should be noted, however, that the absolute Hg concentrations were very low (mean: 0.043 mg·kg^−1^, well below the Class I limit of 0.2 mg·kg^−1^).

The generally low contamination level was further supported by the P_N_ and PLI results (Figure 4b,c). Most sites had P_N_ values well below the safe threshold, indicating a low integrated pollution level. PLI values were mainly distributed between 0.8 and 1.2, suggesting no pollution to slight pollution. Slightly elevated PLI values at a few locations may be related to localized sediment disturbance or episodic anthropogenic inputs [62]. Under this overall low-contamination background, the contamination-factor petal diagram (Figure 4d) showed that Hg (≈1.45) and Pb (≈1.11) were the main relative contributors to the regional pollution load, whereas Zn approached unity and the other metals remained close to background levels. Overall, these indices indicate that heavy metal contamination in the sediments was generally low, with mild enrichment of Hg and Pb. This pattern supports further source apportionment to distinguish anthropogenic inputs from natural background contributions.

### 3.5. Source Apportionment of Heavy Metals

**Figure 5:**
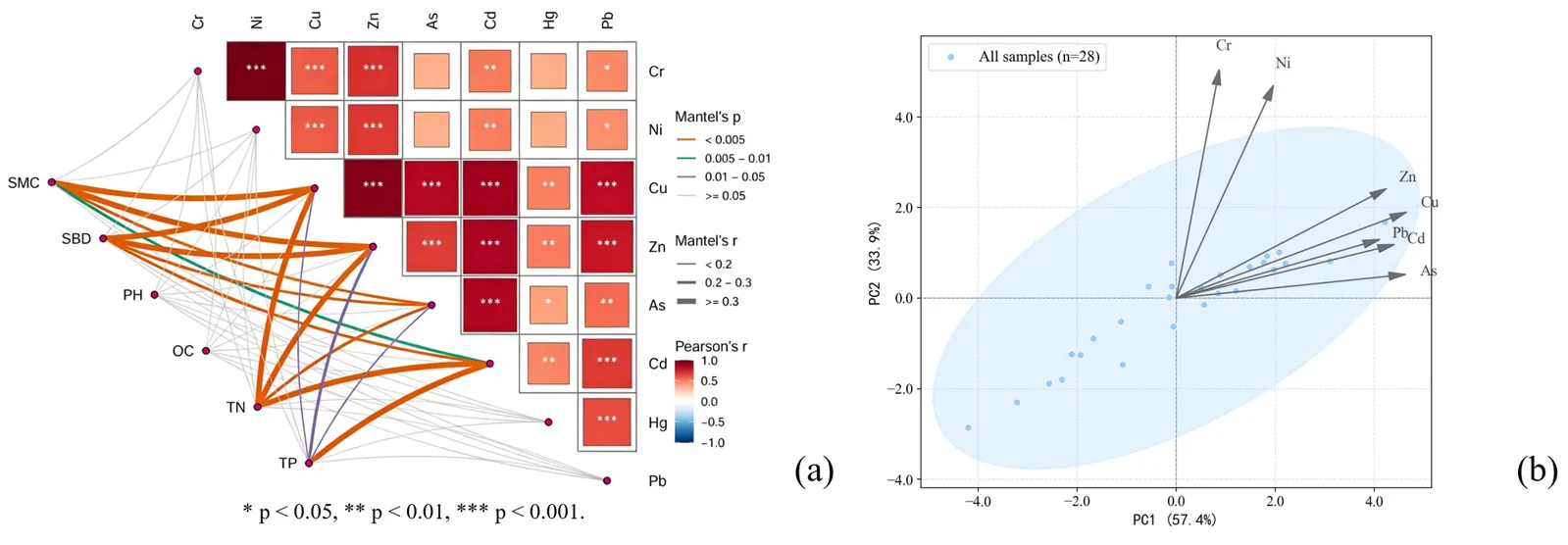
(**a**) Spearman correlation and Mantel test results for heavy metals and sediment physicochemical properties, (**b**) PCA model results of heavy metals in sediments.

**Figure 6:**
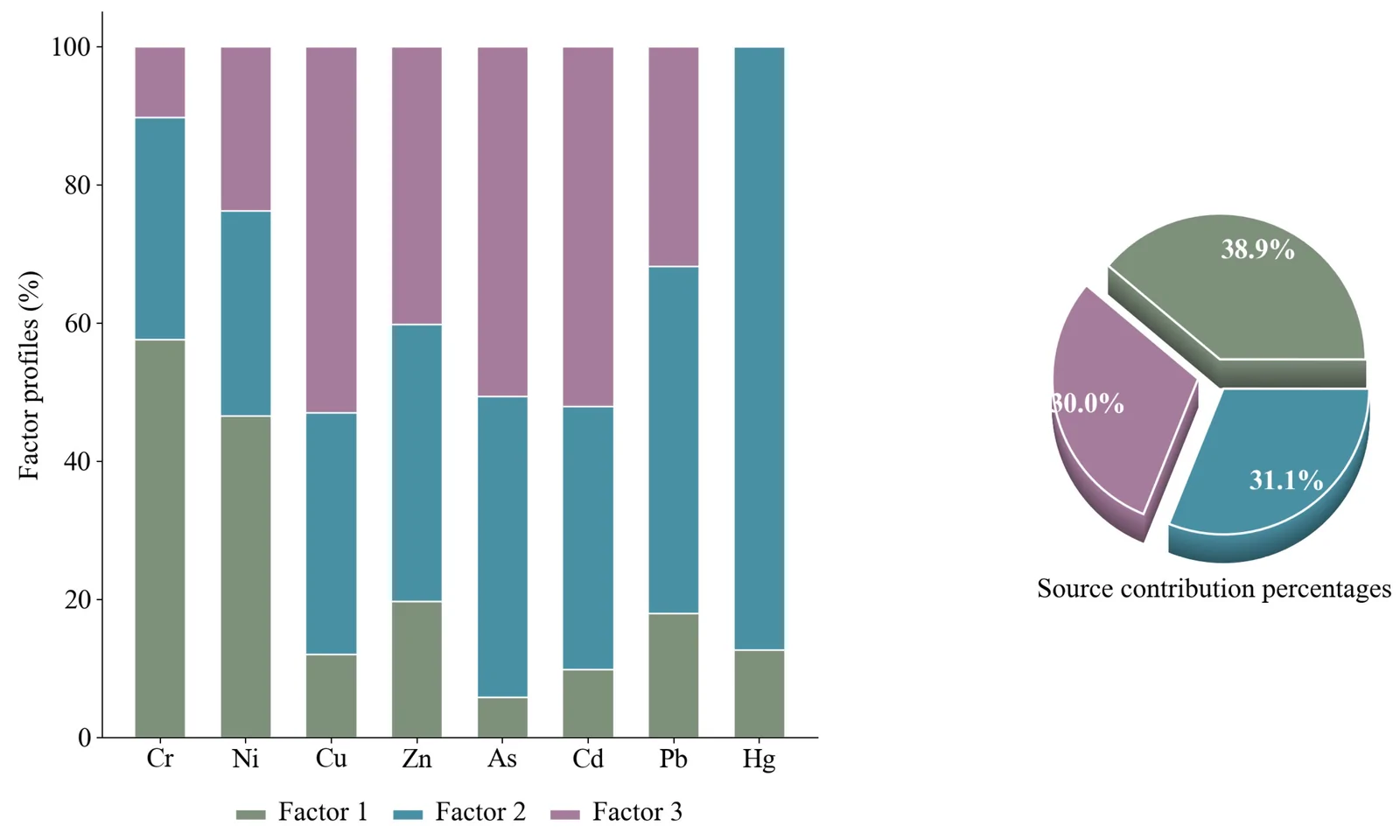
Source profiles and contribution percentages derived from the PMF model.

A combination of Spearman correlation analysis, hierarchical cluster analysis (HCA), and principal component analysis (PCA) was used to qualitatively identify potential sources of heavy metals in the sediments. The three methods produced generally consistent groupings of heavy metals. Spearman correlation analysis (Figure 5) revealed a significant positive correlation between Cr and Ni (*p* < 0.01), while Cu, Zn, Pb, As, and Cd showed strong interelement associations (Spearman’s *p* > 0.8). These associations suggest similar geochemical behaviors or potential common sources within these groups. PCA results further supported this differentiation (Table S8, Figure 6), with Cr and Ni showing high loadings on the second principal component (PC2), which explained 33.88% of the variance. These two elements also clustered together in the HCA dendrogram (Figure S1). Together with their relatively stable spatial distributions, this component was mainly associated with the natural lithogenic background [63]. In contrast, Cu, Zn, Pb, As, and Cd showed high loadings on the first principal component (PC1), which explained 57.37% of the total variance. Combined with the HCA results and the industrial context of the study area, this cluster was likely related to port logistics, transportation, industrial emissions, and other local anthropogenic activities [29,64]. It was therefore interpreted as a composite anthropogenic source. Notably, Hg was excluded from the PCA model because of its low measure of sampling adequacy (MSA = 0.264), which indicated weak covariance with the other metals and helped to improve the robustness of the loading matrix. However, the isolated Hg cluster in the HCA (Figure S1) suggested a source pathway different from the other metals, likely associated with regional coal combustion and atmospheric deposition [56]. Additionally, Mantel tests (Figure 5) showed that total nitrogen (TN), sediment moisture content (SMC), and sediment bulk density (SBD) were the main physicochemical factors associated with heavy metal distributions in the study area (Mantel’s r ≥ 0.3, *p* < 0.005). These results suggest that nutrient conditions and sediment physical properties may influence metal enrichment [65].

Following the qualitative identification, the positive matrix factorization (PMF) model was used to quantify source contributions. Based on the $Q/Q_{exp}$ ratio (0.649), scaled residual distributions, and factor interpretability (Table S9), the three-factor solution was selected as the optimal model. The factor profiles and contribution ratios are shown in Table S10 and Figure 6. Factor 1 accounted for 38.9% of the total contribution and was characterized by high contributions of Cr (57.58%) and Ni (46.52%). This factor primarily reflected parent-material control and mineral weathering and was interpreted as the lithogenic background source [63]. Factor 2 accounted for 31.1% of the total contribution, with Hg (87.35%) as the dominant element, followed by relatively high contributions of Pb (50.25%) and As (43.60%). Given that Hg is a typical indicator of coal combustion, Factor 2 was associated with atmospheric deposition influenced by regional fossil fuel combustion and long-range aerosol transport [56]. Factor 3 contributed 30.0% and was mainly characterized by Cu (52.99%), Cd (52.05%), and As (50.60%). Considering the sampling-site distribution in this study area, this factor likely represented mixed anthropogenic inputs, including port logistics, traffic-related emissions and wear, and minor agricultural inputs [29,64]. Overall, heavy metals in the sediments were jointly influenced by lithogenic background (38.9%), atmospheric deposition (31.1%), and local anthropogenic activities (30.0%). Although the atmospheric-deposition and local anthropogenic factors together accounted for 61.1%, most metal concentrations remained close to or below regional background values. This indicates that human activities have influenced sediment metal composition, but have not yet resulted in severe contamination.

### 3.6. Ecological Risk Assessment

**Figure 7:**
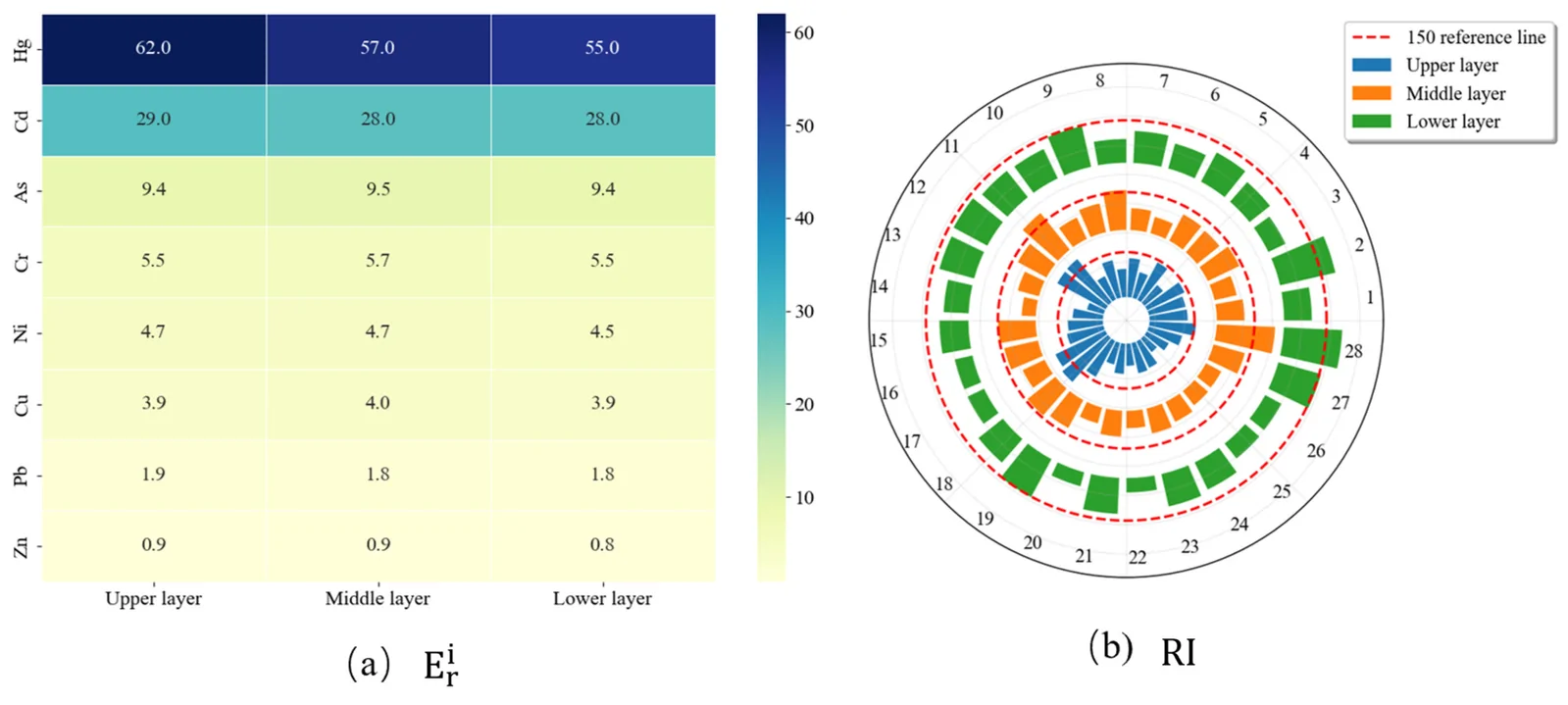
Ecological Risk Assessment Results. (**a**) Average values of the potential ecological risk factor ($E_{r}^{i}$) for each metal across the three sediment layers (Upper, Middle, Lower). (**b**) Integrated ecological risk index (RI) for each sampling site across the three layers.

Ecological risk was evaluated using the potential ecological risk factor ($E_{r}^{i}$) and the integrated risk index (RI) (Figure 7). Although the absolute concentration of Hg was very low (mean: 0.043 mg·kg^−1^), it exhibited the highest $E_{r}^{i}$ values (55.0–62.0), consistently falling within the moderate-risk category and acting as the dominant contributor to ecological risk. This pattern mainly reflects the high toxic-response factor of Hg ($T_{r}^{i}=40$). Cd showed $E_{r}^{i}$ values of approximately 28–29, approaching the moderate-risk threshold and suggesting potential ecological concern. Although As remained within the low-risk range ($E_{r}^{i}\approx 9.4$), its values were higher than those of Cr, Ni, Cu, Zn, and Pb, indicating a relatively greater ecological contribution. The remaining metals showed consistently low ecological risks.

Integrated RI values indicated a generally low ecological risk level across the study area. Across sediment layers, the contribution hierarchy was consistent, with Hg as the dominant contributor followed by Cd. This pattern may reflect the combined effect of source inputs and high toxic-response factors for Hg and Cd. Spatially, most RI values remained below 150, indicating low ecological risk (Figure 7b). However, several stations, such as Sites 27–28 and 1–2, showed relatively higher RI values, reflecting localized heterogeneity consistent with the single-metal risk results.

Linking ecological risk with PMF source apportionment helped clarify the source-related risk patterns. The dominance of Hg was consistent with Factor 2, which was identified as the atmospheric deposition source, and was amplified by its high toxic-response factor ($T_{r}^{i}=40$) [45,66]. The ecological relevance of Cd was more closely linked to Factor 3, which represented mixed local anthropogenic inputs, including minor agricultural influence. Its risk contribution was amplified by the high toxic-response factor of Cd ($T_{r}^{i}=30$), despite its low concentration. In contrast, metals such as Cr, Ni, Cu, Zn, and Pb showed relatively low ecological risk because of their lower toxic-response factors, even when they contributed substantially to the total metal load. This difference between mass contribution and toxicity-weighted risk indicates that both concentration and metal-specific toxicity should be considered in ecological risk assessment [67].

Although the coastal wetlands generally remained at a low ecological risk level, Hg and Cd should receive continued attention because of their relatively higher ecological contributions. These results suggest that ecological risk management should focus on Hg associated with atmospheric deposition and Cd associated with local anthropogenic inputs.

### 3.7. Health Risk Assessment and Source-Risk Pathway Analysis

The probabilistic non-carcinogenic risk (HI) and total carcinogenic risk (TCR) of heavy metals in surface sediments were evaluated using the USEPA health risk model and Monte Carlo simulation with 100,000 iterations. Localized parameters were incorporated to improve regional applicability, including different exposure frequencies for core residential areas (90 d/y), non-core adults (250 d/y), and non-core children (50 d/y), together with inhalation, Cr(VI), and As bioavailability corrections.

The simulation results (Table 3) showed that HI values for all receptor groups were below the safety threshold (HI < 1), indicating no significant non-carcinogenic risk. TCR values were also far below the commonly used unacceptable risk threshold (1 × 10^−4^), suggesting that the overall carcinogenic risk remained within an acceptable range. In core residential areas, mean TCR values ranged from 4.96 × 10^−7^ to 5.81 × 10^−7^, and the probability of TCR exceeding 1 × 10^−6^ remained below 1% for all receptor groups, indicating negligible carcinogenic risk. Children showed higher HI values than adults in core residential areas, mainly because of their lower body weight and higher intake per unit body mass [68].

By contrast, in non-core industrial–agricultural areas, adults showed higher risk levels than children. The mean HI values of adults ranged from 0.216 to 0.239, and their mean TCR values ranged from 1.37 × 10^−6^ to 1.61 × 10^−6^. The probabilities of TCR exceeding 1 × 10^−6^ reached 84.607% for adult males and 94.561% for adult females, indicating that adult receptors in non-core areas entered the acceptable carcinogenic risk range, although their risks remained far below the unacceptable threshold of 1 × 10^−4^. In contrast, children in non-core areas showed lower risk levels, with a mean HI of 0.099, a mean TCR of 3.14 × 10^−7^, and no probability of exceeding 1 × 10^−6^. This pattern suggests that increased exposure frequency in non-core industrial–agricultural areas outweighed the physiological sensitivity of children in determining health risk levels.

**Figure 8:**
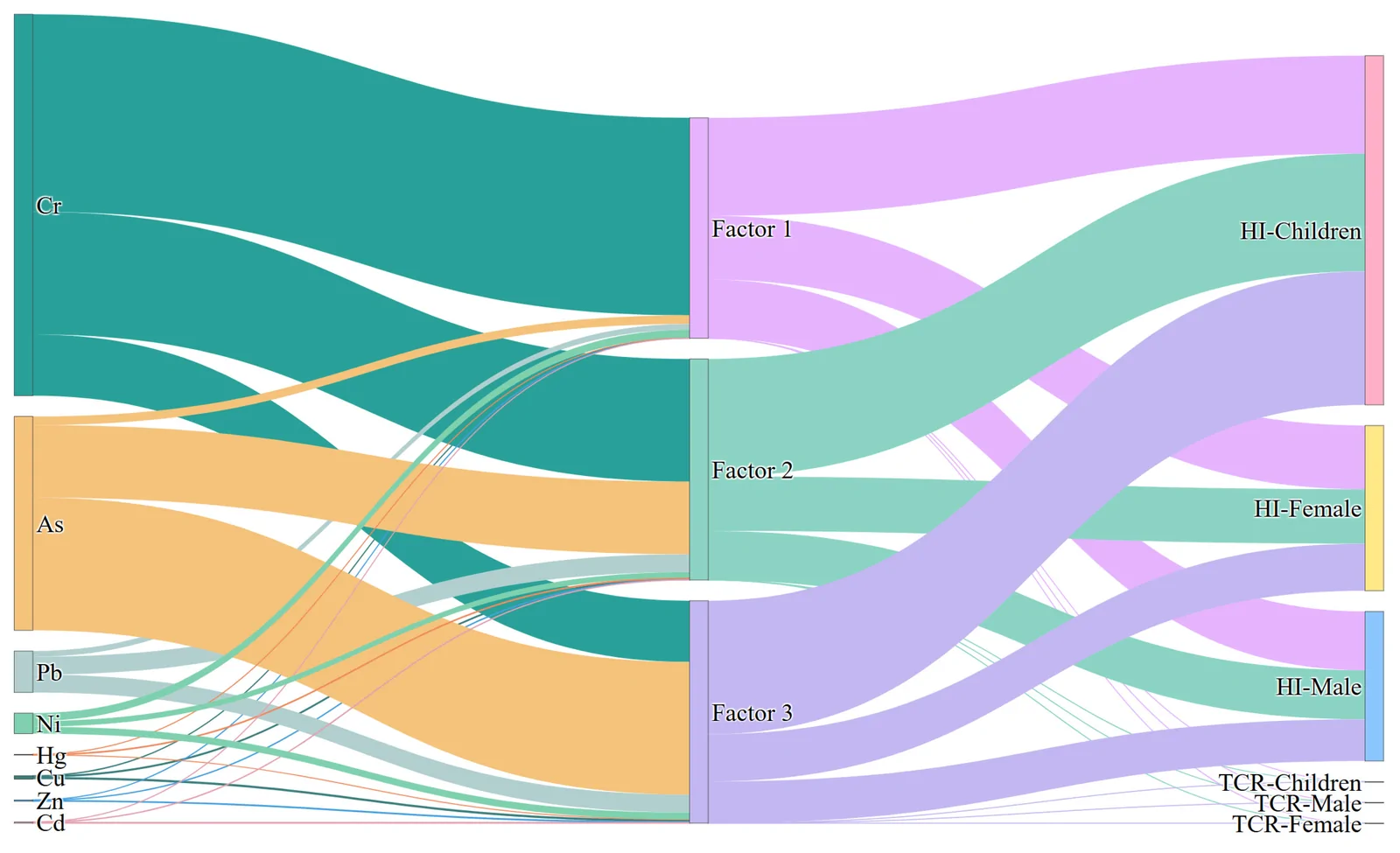
PMF-based source–element–risk pathways of heavy metals in wetland sediments.

To further examine how pollution sources contributed to health risks, a source–element–risk pathway model was developed by integrating PMF apportionment with risk assessment (Figure 8). The pathway model showed that metal concentration contributions and risk contributions were not always consistent. Non-carcinogenic risk was mainly associated with metals with relatively high environmental abundance. Cr, which was mainly derived from the lithogenic background source (Factor 1), made the largest contribution to HI. Although Cr was not the most toxic metal in the health risk model, its relatively high concentration increased its contribution to non-carcinogenic risk. This indicates that HI was largely controlled by environmental abundance rather than toxicity alone.

In contrast, TCR was mainly controlled by toxicity parameters. As was the dominant contributor to carcinogenic risk, primarily because of its high carcinogenic slope factor. Its source contribution was closely associated with the local anthropogenic factor (Factor 3), with additional influence from atmospheric deposition (Factor 2). This result indicates that As can dominate carcinogenic risk even at relatively low concentrations when toxicity parameters are considered. Therefore, local anthropogenic inputs of As should be regarded as a key pathway for carcinogenic risk accumulation.

The pathway model also showed a mismatch between ecological and human health risk contributions. Hg dominated ecological risk because of its high toxic-response factor, but it contributed little to human health risk under the current exposure scenarios. This difference suggests that ecological and human receptors respond differently to metal toxicity and exposure pathways. In other words, elements with high ecological risk do not necessarily dominate human health risk, and source-oriented risk management should distinguish between ecological and human exposure endpoints.

Overall, the source–risk pathway analysis revealed two distinct risk-control patterns. Non-carcinogenic risk was mainly driven by background-derived Cr due to its relatively high environmental abundance, whereas carcinogenic risk was primarily controlled by anthropogenic As inputs because of its high carcinogenic potency. These findings suggest that risk management in the study area should combine long-term monitoring of background-derived Cr with targeted reduction of anthropogenic As inputs. In addition, exposure protection for adults in non-core industrial–agricultural areas should be strengthened to reduce long-term carcinogenic risk accumulation.

## 4. Conclusions

In summary, coastal wetland sediments in the coastal wetlands of western Bohai Bay were characterized by generally low heavy metal contamination, with most metals remaining close to or below regional background values and marine sediment quality limits. The vertical profiles showed no significant inter-layer differences for most metals, indicating a relatively homogeneous sediment column under the influence of tidal disturbance, sediment resuspension, and bioturbation. Pollution assessment further confirmed the low contamination status, although Hg and Pb were the main relative contributors to the regional pollution load. Source apportionment indicated that heavy metals were jointly influenced by lithogenic background (38.9%), atmospheric deposition (31.1%), and local anthropogenic activities (30.0%). Cr and Ni were mainly associated with natural geological sources, Hg was related to regional coal combustion and atmospheric deposition, while Cu, Zn, Pb, Cd, and As were more closely linked to port logistics, transportation, industrial emissions, and minor agricultural inputs. Ecological risk assessment showed that the integrated RI remained at a low level, but Hg, despite its very low absolute concentration (mean: 0.043 mg·kg^−1^), consistently represented the dominant ecological risk contributor due to its high toxic-response factor, and Cd approached the moderate-risk threshold. Human health risks were generally acceptable for all receptor groups, with HI values below 1 and TCR values far below the unacceptable risk threshold. However, adults in non-core industrial–agricultural areas showed relatively higher carcinogenic risks because of increased exposure frequency. The source apportionment combined with risk assessment further showed that non-carcinogenic risk was mainly associated with background-derived Cr, whereas carcinogenic risk was primarily controlled by anthropogenic As inputs. Hg showed a clear mismatch between ecological and human health risks, contributing substantially to ecological risk but only weakly to human health risk under current exposure scenarios. Overall, although the sediments remained under low contamination and acceptable ecological–health risk conditions, long-term monitoring should focus on Hg, Cd, and As, with particular attention to atmospheric deposition, local anthropogenic inputs, and adult exposure in non-core industrial–agricultural areas.

## Figures and Tables

**Table 1:** Statistical summary of sediment physicochemical properties.

Item	SMC (%)	SBD (g·cm^−3^)	pH	OC (g·kg^−1^)	TN (%)	TP (%)
Maximum	0.958	1.81	8.71	20.383	0.163	0.089
Minimum	0.144	0.77	7.70	0.315	0.011	0.036
Mean	0.418	1.35	8.21	7.057	0.062	0.062
SD	0.204	0.27	0.21	4.486	0.032	0.011
Median	0.382	1.37	8.20	6.572	0.062	0.063

**Table 2:** Heavy Metal Content Statistics, mg/kg.

Item	Cr	Ni	Cu	Zn	As	Cd	Hg
Maximum	149.65	50.94	39.00	107.32	15.71	0.22	0.08
Minimum	42.10	17.53	10.56	40.39	5.78	0.07	0.02
Average	74.16	26.94	23.74	71.05	11.33	0.14	0.04
SD	20.62	6.65	7.50	3.07	18.49	0.04	0.02
Median	73.73	25.69	22.80	11.65	70.47	0.15	0.04
CV (%)	27.8	24.7	31.6	26.0	27.1	30.1	31.5
GB 18668-2002 Class I ^a^	80	–	35	150	20	0.5	0.2

Note: ^a^ Class I limit of the Marine Sediment Quality Standard (GB 18668-2002) [54]; background values from Cheng et al. [46].

**Table 3:** Probabilistic human health risks of heavy metals in wetland sediments.

Zone	Receptor	Mean HI	HI_95_	Mean TCR	TCR_95_	p (TCR > 10^−6^)
Core	AM	0.085	0.143	4.96 × 10^−7^	7.19 × 10^−7^	0.030%
	AF	0.094	0.157	5.81 × 10^−7^	8.41 × 10^−7^	0.514%
	CH	0.187	0.282	5.67 × 10^−7^	8.41 × 10^−7^	0.782%
Non-core	AM	0.216	0.282	1.37 × 10^−6^	2.01 × 10^−6^	84.607%
	AF	0.239	0.311	1.61 × 10^−6^	2.36 × 10^−6^	94.561%
	CH	0.099	0.136	3.14 × 10^−7^	4.70 × 10^−7^	0.000%

## Data Availability

The original contributions presented in this study are included in the article/Supplementary Material. Further inquiries can be directed to the corresponding authors.

## Supplementary Materials

The following supporting information can be downloaded at: https://www.mdpi.com/article/10.3390/toxics14080654/s1, Figure S1: Hierarchical cluster analysis dendrogram of heavy metals; Table S1: Classification criteria for heavy metal pollution indices; Table S2: Classification criteria for potential ecological risk factor ($E_{r}^{i}$) and integrated ecological risk index (RI); Table S3: Parameters for Human Health Risk Assessment; Table S4: Toxicity parameters and correction factors for human health risk assessment; Table S5: Comparison of heavy metal concentrations in surface sediments from different coastal regions (mg·kg^−1^, mean values); Table S6: Layer-averaged concentrations and inter-layer differences of heavy metals; Table S7: Layer-specific coefficients of variation (CVs) for heavy metals; Table S8: Principal component loading matrix of heavy metals; Table S9: Comparison of model diagnostics and evaluation results for different numbers of factors; Table S10: Characteristics and source interpretation of PMF factors.
