# Supplementary material for: Integrating Vertical Distribution, Quantitative Source Apportionment, and Source-Oriented Risk Assessment of Heavy Metals in Coastal Wetland Sediments: A Case Study from the Western Bohai Bay Watershed
Source: Toxics. 2026 Jul 25;14(8):654. doi: 10.3390/toxics14080654 (PMC13517330; doi:10.3390/toxics14080654)
Supplement: Supplementary file 1 [file toxics-14-00654-s001.zip › toxics-4433896-supplementary.pdf]

Table S1. Classification criteria for heavy metal pollution indices.

Index	Value Range	Class/Grade	Contamination Level
Geoaccumulation Index (Igeo)	Igeo ≤ 0	0	Unpolluted
	0 < Igeo ≤ 1	1	Unpolluted to moderately polluted
	1 < Igeo ≤ 2	2	Moderately polluted
	2 < Igeo ≤ 3	3	Moderately to heavily polluted
	3 < Igeo ≤ 4	4	Heavily polluted
	4 < Igeo ≤ 5	5	Heavily to extremely polluted
	Igeo > 5	6	Extremely polluted
Nemerow Pollution Index (PN)	PN ≤ 0.7	-	Clean (Unpolluted)
	0.7 < PN ≤ 1.0	-	Warning limit
	1.0 < PN ≤ 2.0	-	Moderately polluted
	PN > 2.0	-	Heavily polluted
Pollution Load Index (PLI)	PLI ≤ 1	-	Unpolluted
	1 < PLI ≤ 2	-	Slightly polluted
	2 < PLI ≤ 3		Moderately polluted
	PLI > 3		Heavily polluted

Table S2. Classification criteria for potential ecological risk factor (E_r^i) and integrated ecological risk index (RI).

Single heavy metal		Multiple heavy metals	
E_r^i	Risk level	RI	Risk level
≤40	Low risk	≤150	Low risk
(40, 80]	Moderate risk	(150, 300]	Moderate risk
(80, 160]	High risk	(300, 600]	High risk
(160, 320]	Severe risk	＞600	Severe risk
＞320	Extremely high risk	——	——

Table S3. Parameters for Human Health Risk Assessment

Parameter	Description	Unit	Adult male (AM)	Adult female (AF)
BW	Body weight	kg	N(67.55, 6.00)	N(57.59, 5.00)
IRs	Soil ingestion rate	mg/d	N(100.00, 15.00)	N(100.00, 15.00)
SA	Exposed skin area	cm^2	N(5400.00, 400.00)	N(4900.00, 350.00)
IRi	Inhalation rate	m^3/d	N(19.00, 2.00)	N(14.00, 1.50)
EF_core	Exposure frequency in core residential area	d/y	90	90
EF_non-core	Exposure frequency in non-core industrial–agricultural area	d/y	250	250
ED	Exposure duration	y	24	24
AF	Skin adherence factor	$mg/(cm^2 \cdot d)$	0.2	0.2
ABS	Dermal absorption factor	dimensionless	0.001	0.001
PEF	Particle emission factor	m^3/kg	1.36×10^9	1.36×10^9
AT_nc	Averaging time for non-carcinogenic risk	d	365	365
AT_c	Averaging time for carcinogenic risk	d	70 × 365	70 × 365

Note: N(μ, σ) indicates a normal distribution with mean μ and standard deviation σ. In the Monte Carlo simulation, BW, IRs, SA, and IRi were randomly sampled from normal distributions and truncated to positive values. EF values differed between the core residential area and the non-core industrial–agricultural area.

Table S4. Toxicity parameters and correction factors for human health risk assessment.

Metal	RfD_ing	RfD_der	SF_ing	SF_der	SF_inh
Cr	0.003	0.000006	0.0085	0	42.0
Ni	0.020	0.000060	0	0	0
Cu	0.040	0.012000	0	0	0
Zn	0.300	0.060000	0	0	0
As	0.0003	0.000300	1.5	3.66	15.1
Cd	0.001	0.000010	6.1	0	6.3
Pb	0.0035	0.000525	0.0085	0	0
Hg	0.0003	0.000021	0	0	0

Table S5. Comparison of heavy metal concentrations in surface sediments from different coastal regions (mg·kg^{-1}, mean values).

Parameter	Cr	Ni	Cu	As	Zn	Cd	Hg	Pb
2022 Hangzhou Bay	60.40	-	33.34	10.53	92.63	0.14	0.05	21.10
2015 Bohai Bay	60.47	-	23.18	16.09	87.22	0.23	0.03	38
2016 Haizhou Bay	76.38	-	31.95	11.67	78.31	0.03	0.03	28.00

Note: All data are mean concentrations reported in the respective studies. "–" indicates data not available.

Table S6. Layer-averaged concentrations and inter-layer differences of heavy metals.

metal	$C_{0–20}$	$C_{20–40}$	$C_{40–60}$	ΔC mid–surf	ΔC mid–bottom
Cr	75.89	74.71	71.88	-1.18	2.83
Ni	32.69	32.84	31.47	0.16	1.37
Cu	23.55	24.01	23.67	0.46	0.34
Zn	71.81	71.57	69.76	-0.24	1.82
As	11.3	11.45	11.25	0.15	0.2
Cd	0.144	0.14	0.139	-0.0047	0.0004
Hg	0.0466	0.043	0.0409	-0.0036	0.0021
Pb	27.4	28.26	27.27	0.86	0.99

Table S7. Layer-specific coefficients of variation (CVs) for heavy metals.

Heavy Metal	Upper Layer CV	Middle Layer CV	Lower Layer CV
Cr	35.10%	24.40%	29.30%
Ni	33.60%	25.20%	29.60%
Cu	34.60%	32.50%	34.10%
Zn	29.30%	25.90%	28.10%
As	29.80%	26.60%	30.10%
Cd	37.20%	31.30%	33.00%
Hg	49.70%	50.80%	51.30%
Pb	30.10%	47.70%	35.70%

Table S8. Principal component loading matrix of heavy metals.

Metal	PC1	PC2
Cr	0.17	1.00
Ni	0.39	0.93
Cu	0.92	0.38
Zn	0.84	0.48

As	0.92	0.10
Cd	0.87	0.23
Pb	0.82	0.26
Eigenvalue	4.02	2.37
Variance explained (%)	57.37	33.88
Cumulative variance explained (%)	57.37	91.25

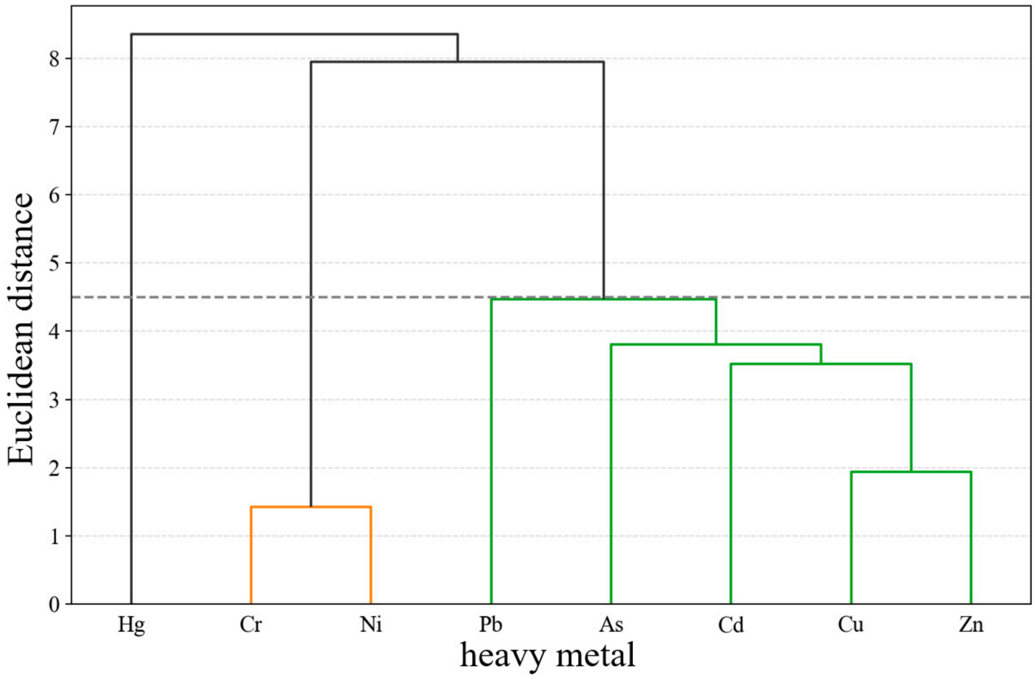

Figure S1. Hierarchical cluster analysis dendrogram of heavy metals.

Table S9. Comparison of model diagnostics and evaluation results for different numbers of factors

Factor solution	Q/Qexp	Scaled residual range	Residuals within ±3	Base runs convergence	Model assessment
2-factor	1.017	−2.605 to 5.364	222/224	20/20	Insufficient source separation
3-factor	0.649	−1.647 to 2.135	224/224	20/20	Optimal and interpretable solution
4-factor	0.492	−1.559 to 2.036	224/224	20/20	Possible overfitting and factor splitting

Table S10. Characteristics and source interpretation of PMF factors.

Factor	Characteristic Elements	Element Contribution Characteristics	Source Interpretation

Factor 1	Cr, Ni	Cr (57.58%) and Ni (46.52%) mainly originate from this factor, with relatively high contributions within the factor	Natural background source (parent material control)
Factor 2	Hg, Pb, Zn, As	Hg (87.35%) is dominant, with high contributions from Pb (50.25%), Zn (40.03%), and As (43.60%)	Atmospheric deposition – mixed traffic and port source
Factor 3	Cu, Cd, As, Zn	Cu (52.99%), Cd (52.05%), and As (50.60%) show significant contributions	Industrial–agricultural mixed anthropogenic source
